# The Influence of Alcohol Consumption in Conjunction with Sex Hormone Deficiency on Ca/P Ratio in Rats

**DOI:** 10.1155/2016/3797139

**Published:** 2016-03-17

**Authors:** Karina Bortolin Lodi, Adriana Mathias Pereira da Silva Marchini, Ana Maria do Espírito Santo, Sigmar de Mello Rode, Leonardo Marchini, Rosilene Fernandes da Rocha

**Affiliations:** ^1^Institute of Science and Technology, Department of Biosciences and Diagnostics, Universidade Estadual Paulista (UNESP), Avenida Engenheiro Francisco José Longo 777, 12245-000 São José dos Campos, SP, Brazil; ^2^Institute of Environmental Science, Chemical and Pharmaceutical, Department of Mathematical and Earth Sciences, Federal University of São Paulo (UNIFESP), Rua Prof. Artur Riedel 275, 09972-270 Diadema, SP, Brazil; ^3^Institute of Research and Development, University of Vale do Paraíba (UNIVAP), Laboratory of Biomedical Vibrational Spectroscopy, Avenida Shishima Hifumi 2911, 12244-000 São José dos Campos, SP, Brazil; ^4^Institute of Science and Technology, Department of Dental Materials and Prosthesis, Universidade Estadual Paulista (UNESP), Avenida Engenheiro Francisco José Longo 777, 12245-000 São José dos Campos, SP, Brazil; ^5^Department of Preventive and Community Dentistry, The University of Iowa College of Dentistry and Dental Clinics, N337-1 Dental Science N, 52242 Iowa City, IA, USA

## Abstract

Deficiency of sex hormones and excessive alcohol consumption are factors that have been related to alterations in the pattern of bone mineralization and osteoporosis. The aim of this study was to evaluate possible alterations in the calcium/phosphorus (Ca/P) ratio in the femur of rats subjected to sex hormone deficiency and/or alcohol consumption.* Methods*. Female and male Wistar rats (*n* = 108) were divided into ovariectomized (Ovx), orchiectomized (Orx), or sham-operated groups and subdivided according to diet: alcoholic diet (20% alcohol solution), isocaloric diet, and* ad libitum* diet. The diets were administered for 8 weeks. The Ca/P ratio in the femur was analyzed by energy dispersive micro-X-ray spectrometer (*μ*EDX).* Results*. Consumption of alcohol reduced the Ca/P ratio in both females and males. The isocaloric diet reduced the Ca/P ratio in females. In groups with the* ad libitum* diet, the deficiency of sex hormones did not change the Ca/P ratio in females or males. However, the combination of sex hormone deficiency and alcoholic diet presented the lowest values for the Ca/P ratio in both females and males.* Conclusions*. There was a reduced Ca/P ratio in the femur of rats that consumed alcohol, which was exacerbated when combined with a deficiency of sex hormones.

## 1. Introduction

Bone is a specialized form of connective tissue formed by minerals, organic matrix, water, and lipids. Calcium (Ca) and phosphorus (P) are the most important constituent elements of the bone mineral phase, forming hydroxyapatite crystals [Ca_10_(PO_4_)_6_(OH)_2_]. Compared with geological hydroxyapatite, the bone crystals are small, poorly crystalline, and more soluble. This solubility allows the maintenance of mineral homeostasis, since the Ca and P also have important metabolic functions [1, 2]. In adults, the degree of mineralization depends, primarily, on changes in the bone remodeling process. This process is characterized by constant activity of osteoclasts (bone resorbing cells) and osteoblasts (bone forming cells). Diseases that change the pattern of bone turnover, such as osteoporosis, can modify the degree of bone mineralization as well as the chemical composition of the hydroxyapatite, changing Ca and P proportions [3, 4].

Osteoporosis can be classified as primary and secondary. In clinical practice, the most common forms of primary osteoporosis are those that affect women after menopause (type I) and age-related osteoporosis that affects both women and men (type II) [5, 6].

Deficiency of sex hormones has an important role in the development of osteoporosis. This deficiency is well-known to affect women after menopause. However, osteoporosis caused by sex hormone deficiency can also affect men. In males, the serum levels of testosterone (the main sex steroid regulating bone metabolism in men) gradually decrease with age. This leads to decreased levels of estrogen (since estrogen can be synthesized from testosterone). Both estrogen and testosterone are important in maintaining bone health; however, estrogen deficiency seems to be the most important determinant of bone loss associated with age in both men and women [7–10]. It is believed that estrogen deficiency causes changes in bone remodeling due primarily to an increase in the resorption process, although a decrease in bone formation is also described [6, 10, 11].

Osteoporosis can also present secondary causes related mainly to lifestyle, nutritional abnormalities, use of medicines, and endocrine/metabolic disorders. Secondary causes of osteoporosis could increase bone loss and fracture risk, especially in the elderly [5, 6]. Chronic abuse of alcohol is also widely cited as a cause of secondary osteoporosis [12, 13]. The decrease in bone mass related to the consumption of alcohol seems to be related to bone remodeling alterations, with a predominant decrease in bone formation, as alcohol has been considered toxic for osteoblasts [13, 14].

Alcohol consumption can also cause significant nutritional changes. In part, this happens because alcohol has low nutritional value but high energy content, which may lead the consumer to replace the intake of nutritive food with alcohol. Moreover, consumption of alcohol leads to malabsorption and maldigestion of food [15]. Since adequate nutrient intake is important for maintaining bone quality [16], this factor should be considered in studies aiming to evaluate the influence of alcohol on bone tissue. Alcohol consumption is also related to cognitive disorders that lead to an increased incidence of falls and bone fractures, which can increase levels of morbidity and mortality in patients with osteoporosis [17].

Considering the importance of Ca and P on bone chemical composition, and the negative influence of estrogen/testosterone deficiency and/or alcohol consumption on bone quality, the objective of this study is to evaluate possible changes in the quantity of Ca and P in the femur of ovariectomized (Ovx) or orchiectomized (Orx) rats receiving an alcoholic diet.

In previously published studies, adverse effects on bone quality were observed in female rats when alcohol was associated with estrogen deficiency [18, 19], although few data exist about what occurs in males. Based on the results of these studies, our hypothesis is that deficiencies in sex hormones associated with alcohol consumption can cause a change in bone mineralization with a reduced Ca/P ratio.

## 2. Methods

### 2.1. Surgical Procedures, Diet, and Weight

This study was performed in accordance with the ethical standards adopted in animal experimentation and was approved by the Ethics Committee of São José dos Campos School of Dentistry, Universidade Estadual Paulista (UNESP) (protocol number 037/2007-PA/CEP).

One hundred and eight rats (*Rattus norvegicus*, albino, Wistar) at a 1 : 1 male/female ratio, aged four months, were initially divided into Ovx, Orx, or sham-operated (Sham) groups. In the Ovx group, the ovaries were removed; in the Orx group, the testicles were removed. The Sham groups were subjected to a surgical procedure similar to the other groups in terms of anesthesia and surgically exposing the ovaries or testicles, but the ovaries or testicles were not removed. The purpose of this procedure was to control for the possible influence of surgical stress on the results. With this method, the Ovx and Orx groups were subjected to a deficiency of sex hormones and the Sham groups were the experimental control.

One month after the surgery, animals were subdivided into alcoholic, isocaloric, and* ad libitum* dietary groups. The interval of one month between the surgical procedure and the beginning of the diet allows sufficient time for the animal to recover from the stress of the surgical procedure, allowing a good adaptation to dietary changes. This same interval was previously described in other studies [19–22]. In the alcoholic diet group, animals received a 20% alcohol solution as the only source of liquids (freely, until they were satiated) and a commercial solid food (freely, until they were satiated). In the isocaloric diet group, animals received water (freely, until they were satiated), 26.6% sucrose solution (in restricted quantity), and commercial solid food (in restricted quantity). The amount of sucrose solution and solid food offered to animals that received the isocaloric diet contained the same amount of calories ingested by animals with the alcoholic diet the day before (pair-fed diet). In doing this, the animals with the isocaloric diet started the diet a day after the animals with the alcoholic diet. In the* ad libitum* diet, the animals received water (freely, until they were satiated) and commercial solid food (freely, until they were satiated).

In summary, the animals were divided into 12 experimental groups with 9 animals in each (*n* = 9) as described in Table 1. More details regarding the methodology of treatment of animals have been previously published [19]. In this study [19], the authors used the same experimental protocol and treatment group subdivision as described herein, but only for female rats: deficiency of sex hormones (Ovx) associated with alcoholic diet (20% alcohol solution), isocaloric diet, or* ad libitum* diet.

For all groups, the average values of daily intake for solid foods and liquids were recorded. The weight changes (%) were also measured for each animal using the difference between final weight (g) and initial weight (g). The value obtained was then divided by the initial weight and multiplied by 100. The initial weight was measured on day of surgery (Ovx, Orx, or Sham) and the final weight on the day of sacrifice.

To analyze the capacity to convert the energy consumed into body weight, a calculation of the feed efficiency ratio was performed for each animal: the weight gain (g) was divided by total dietary intake (kcal) and multiplied by 100.

At the beginning of dietary treatment, which began one month after surgery, animals with the alcoholic diet underwent an adaptation period of 9 days, in which they received alcohol solutions at gradually increasing concentrations: 3 days receiving 5% alcohol solution, 3 days receiving 10% alcohol solution, and 3 days receiving 15% alcohol solution. This period of adaptation decreases a possible aversion to sudden dietary change, allowing greater acceptance of treatment. A similar adaptation protocol (the same timeframe and alcohol concentrations) has been described previously [20].

After this adaptation period, the alcohol groups began to receive the alcoholic solution at 20%, and the isocaloric groups began to receive the calorie-restricted diet. For the* ad libitum* groups there were no changes in the diet. These diets were maintained for 8 weeks. The daily measurement of the amount of food and liquid intake (to calculate caloric intake, feed efficiency, and water consumption) began only after the 20% concentration had been established. At the end of dietary treatment, the animals were sacrificed.

All animals were anesthetized before surgical procedures (Ovx, Orx, or Sham) and sacrifices. An intramuscular injection of a xylazine chloride (2.3 g/100 mL) and ketamine chloride (1.16 g/10 mL) solution was used for this purpose. Initially, a master solution was prepared with 0.8 mL of xylazine chloride and 0.5 mL of ketamine chloride. To promote general anesthesia, the animals received 0.1 mL of this solution for each 100 g of body weight.

### 2.2. Energy Dispersive Micro-X-Ray Fluorescence Spectroscopy

The right femurs were dissected and stored in a freezer (−80°C). They were evaluated by an energy dispersive micro-X-ray spectrometer (*μ*EDX) to determine the concentration of Ca (%) and P (%), enabling the calculation of the Ca/P ratio. The analyses were performed in equipment with a rhodium X-ray tube (Model 1300 Shimadzu®, Kyoto, Japan). The radiation count was made by a silicon lithium semiconductor detector cooled by liquid nitrogen and connected to a computer for data processing.

The X-ray fluorescence spectroscopy was performed as a semiquantitative analysis. A similar methodology was previously published [23, 24]. The present study fixed the fundamental parameters for Ca and P using commercial stoichiometric hydroxyapatite (Aldrich, synthetic Ca_10_(PO_4_)_6_(OH)_2_, grade 99.999%, lot 10818H). The chemical elements carbon (C), oxygen (O), and hydrogen (H) were set as balance (since the bone tissue consists of a carbonated hydroxyapatite). The total mass proportion of the sample (100%) was calculated as *x* + *y* + *z* (where *x* = Ca, *y* = P, and *z* = C + O + H). Only Ca and P were directly measured by the equipment. The organic elements (C, O, and H) were not directly measured because the equipment used in this study does not perform data for elements below sodium (Na) in atomic number. Other inorganic elements as magnesium (Mg), Na, and potassium (K) were not identified in any spectra, indicating they were absent in the samples or their amounts were below the limit of detection.

Equipment voltage was set at 15 kV with automatic adjustment of the current and beam diameter of 50 *μ*m. The equipment allows drawing a line over which the sample can be analyzed. In this study, the analysis was performed on a horizontal line, which was drawn across the longitudinal length of the bone in the central part (excluding the epiphyses, which were previously cut). The chosen surface is relatively flat, which is important for a proper analysis when using this type of technique. The analyses were performed each 50 *μ*m of bone length, totaling 200 points per sample with a reading time of 10 seconds/real time per point.

After finishing the reading, a graph was generated with the positioning and the mean of the Ca and P concentrations (%). The ratio of the chemical elements in the sample was calculated as the ratio of the relative weight given in the readings of the device, which converts the relative intensity of the characteristic transition energy into element content by weight percentage.

For this study, we considered that the synthetic hydroxyapatite, Ca_10_(PO_4_)_6_(OH)_2_, consists of about 39.90% of Ca, 18.50% of P, 41.41% of O, and 0.20% of H (these values were obtained by stoichiometric calculations from the number of atoms and atomic mass of each chemical element present in the molecular formula of hydroxyapatite). Calculations of Ca/P ratio were made considering the number of atoms of Ca and P (10 and 6, resp.) and the relative percentages of Ca and P (39.90% and 18.50%, resp.) present in the hydroxyapatite molecule, using the following formula:

(1) $$\begin{array}{c} \frac{Ca}{P}=\frac{\left(10\times c\right)÷39.90}{\left(6\times p\right)÷18.50}. \end{array}$$

In the above formula, the letter *c* represents the value of Ca (%) and the letter *p* represents the value of P (%). The values of Ca (%) and P (%) were obtained in the analysis of the samples.

### 2.3. Statistical Analysis

Dietary analysis and feed efficiency were tabulated and presented. Data related to the analysis of Ca (%), P (%), and Ca/P were evaluated by one-way ANOVA and Tukey's test. Data related to changes in weight (%) showed nonnormal distribution and were performed using nonparametric test (Kruskal-Wallis followed by Dunn-Bonferroni). A significance level of 0.05 (5%) was adopted for all tests.

The program Minitab® (version 15.1.1.0 Minitab Inc., State College, PA, USA) was used for statistical analysis. The figures were made using the program Microsoft Excel 2007 (Microsoft Corporation, Redmond, WA, USA).

## 3. Results

### 3.1. Overall Remarks

In animals subjected to the alcoholic diet, a lethargic state was observed, characterized by excessive resting (probably as a result of alcohol consumption). In the Ovx and Orx groups, the atrophy of the uterus and seminal vesicles was noticed after sacrifice.

### 3.2. Weight Changes (%)

**Figure 1:**
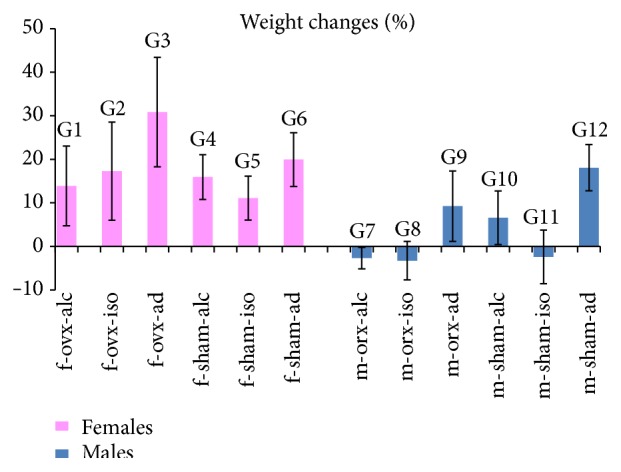
Weight changes (%): the graph illustrates the values of the averages (bars) and standard deviations (lines superimposing the bars). Statistical comparisons are summarized as follows: females: comparison between most of the groups showed no significant differences (NS). Statistical significances (*p* < 0.05) (*∗*) were observed in the following groups: G1/G3 (*∗*); G2/G3 (*∗*); G4/G3 (*∗*); G5/G3 (*∗*). Males: comparison between most of the groups showed no significant differences (NS). Statistical significances (*p* < 0.05) (*∗*) were observed in the following groups: G9/G7 (*∗*); G9/G8 (*∗*); G9/G11 (*∗*); G12/G7 (*∗*); G12/G8 (*∗*); G12/G11 (*∗*).

The values of the averages and standard deviations for weight changes (%) are illustrated in Figure 1. The statistical comparisons for weight changes (%) are summarized in the legend of Figure 1.

In the analysis of weight changes (%), it was observed that all females gained weight during the experiment. The group which gained more weight was G3, representing females with estrogen deficiency and* ad libitum* diet. This group (G3) was statistically different from groups G1 (f-ovx-alc), G2 (f-ovx-iso), G4 (f-sham-alc), and G5 (f-sham-iso). This means that group G3 (f-ovx-ad) was statistically different from all groups with controlled diets (alcoholic or isocaloric).

Regarding the males, animals in groups G7 (m-orx-alc), G8 (m-orx-iso), and G11 (m-sham-iso) lost weight during the experiment. Conversely, males in the other groups gained weight. Males who gained most weight were those with the* ad libitum* diet (G9 and G12). These groups (G9 and G12) were statistically different from G7 (m-orx-alc), G8 (m-orx-iso), and G11 (m-sham-iso).

### 3.3. Caloric Intake, Feed Efficiency, and Water Consumption

For females, the highest values of caloric intake (66.19 kcal) and feed efficiency (46.61%) were observed in G3 (f-ovx-ad). On the other hand, the lowest values of caloric intake (57.76 kcal) and feed efficiency (19.22%) were observed in G5 (f-sham-iso). It is interesting to observe that, in females on an alcoholic diet, G1 (f-ovx-alc) and G4 (f-sham-alc), relatively high caloric intake (64.31 kcal and 65.70 kcal) but relatively low feed efficiency (21.61% and 24.25%) was observed (Table 2).

Regarding males, feed efficiency values were proportional to calorie intake for all groups: when caloric intake increased, the feed efficiency has also increased. The highest values of caloric intake (105.83 kcal) and feed efficiency (17.08%) were found in G12 (m-sham-ad). On the other hand, the lowest values of caloric intake (69.32 kcal) and feed efficiency (−4.73%) were found in G8 (m-orx-iso) (Table 2).

For females, the lowest water consumption occurred in animals receiving the alcoholic diet, G1 (12.99 mL) and G4 (13.71 mL). Similarly, for males, the lowest water consumption also occurred in animals receiving the alcoholic diet, G7 (16.57 mL) and G10 (15.70 mL) (Table 2). It should be emphasized that the calculations for water consumption for animals receiving the alcoholic diet considered the water that the alcohol was dissolved in, and these animals did not receive another form of hydration.

### 3.4. Analysis of Ca (%)

**Figure 2:**
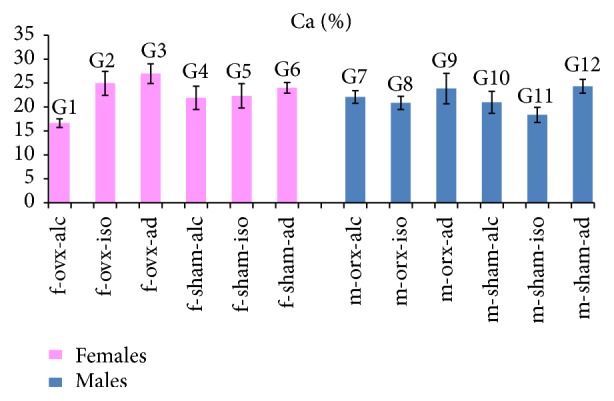
Ca (%): the graph illustrates the values of the averages (bars) and standard deviations (lines superimposing the bars). Statistical comparisons are summarized as follows: females: comparison between most of the groups showed statistical significance (*p* < 0.05). No significant differences (NS) were observed in the following groups: G2/G6 (NS); G4/G5 (NS). Males: comparison between most of the groups showed statistical significance (*p* < 0.05). No significant differences (NS) were observed in the following groups: G9/G12 (NS); G10/G7 (NS); G10/G8 (NS).

The values of the averages and standard deviations for Ca (%) are illustrated in Figure 2. The statistical comparisons for Ca (%) are summarized in the legend of Figure 2.

Regarding females, the highest value for Ca (%) was obtained in G3 (f-ovx-ad), and the lowest value was obtained in G1 (f-ovx-alc). It is interesting to note that both groups (G3 and G1) were submitted to hormonal deficiency, so the difference was in the type of the diet (*ad libitum* or alcoholic). Statistically significant differences were observed among most of the females groups. However, no difference was observed between the following groups: G2 (f-ovx-iso) and G6 (f-sham-ad); G4 (f-sham-alc) and G5 (f-sham-iso).

Regarding males, the highest value for Ca (%) was obtained in G12 (m-sham-ad), and the lowest value was obtained in G11 (m-sham-iso). It is interesting to note that neither group (G12 and G11) had hormone deficiency, so the difference was the type of diet (*ad libitum* or isocaloric). Statistical differences were observed among most of the males groups. However, no difference was observed between the following groups: G9 (m-orx-ad) and G12 (m-sham-ad); G10 (m-sham-alc) and G7 (m-orx-alc); G10 (m-sham-alc) and G8 (m-orx-iso).

### 3.5. Analysis of P (%)

**Figure 3:**
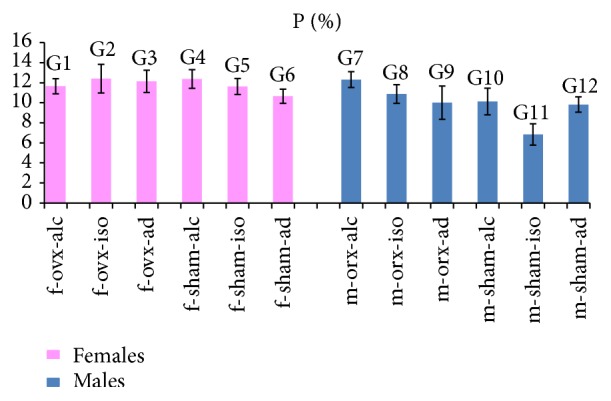
P (%): the graph illustrates the values of the averages (bars) and standard deviations (lines superimposing the bars). Statistical comparisons are summarized as follows: females: comparison between most of the groups showed statistical significance (*p* < 0.05). No significant differences (NS) were observed in the following groups: G1/G5 (NS); G2/G3 (NS); G2/G4 (NS), G3/G4 (NS). Males: comparison between most of the groups showed statistical significance (*p* < 0.05). No significant differences (NS) were observed between the following groups: G10/G12 (NS); G9/G10 (NS); G9/G12 (NS).

The values of the averages and standard deviations for P (%) are illustrated in Figure 3. The statistical comparisons for P (%) are summarized in the legend of Figure 3.

Regarding females, the highest value for P (%) was obtained in G2 (f-ovx-iso), and the lowest value was obtained in G6 (f-sham-ad). Statistically significant differences were observed among most of the females groups. However, no difference was observed between the following groups: G1 (f-ovx-alc) and G5 (f-sham-iso); G2 (f-ovx-iso) and G3 (f-ovx-ad); G2 (f-ovx-iso) and G4 (f-sham-alc); G3 (f-ovx-ad) and G4 (f-sham-alc).

Regarding males, the highest value for P (%) was obtained in G7 (m-orx-alc), and the lowest value was obtained in G11 (m-sham-iso). Statistically significant differences were observed among most of the males groups. However, no difference was observed in the comparisons between the following groups: G10 (m-sham-alc) and G12 (m-sham-ad); G9 (m-orx-ad) and G10 (m-sham-alc); G9 (m-orx-ad) and G12 (m-sham-ad).

It is interesting to note that males subjected to hormonal deficiency and alcoholic diet (G7) demonstrated the highest values of P (%), compared to other groups of males. In contrast, of females subjected to the comparable experimental protocol, G1 (f-ovx-alc) showed a small reduction in P (%) relative to most of the other female groups.

### 3.6. Analysis of Ca/P Ratio

**Figure 4:**
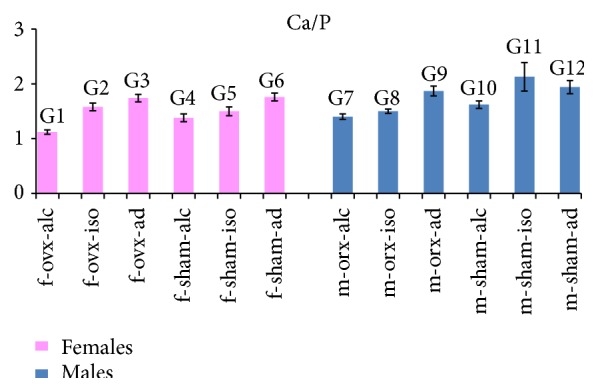
Ca/P: the graph illustrates the values of the averages (bars) and standard deviations (lines superimposing the bars). Statistical comparisons are summarized as follows: females: comparison between most of the groups showed statistical significance (*p* < 0.05). No significant differences (NS) were observed in the following groups: G3/G6 (NS). Males: comparison between most of the groups showed statistical significance (*p* < 0.05). No significant differences (NS) were observed in the following groups: G12/G9 (NS); G12/G11 (NS).

The values of the averages and standard deviations for Ca/P are illustrated in Figure 4. The statistical comparisons for Ca/P are summarized in the legend of Figure 4.

For females, statistically significant differences in Ca/P were observed among most of the experimental groups. No statistical differences were found only between G3 (f-ovx-ad) and G6 (f-sham-ad), representing groups with* ad libitum* diets, which also demonstrated higher values of Ca/P.

For males, statistically significant differences were also observed among most of the experimental groups. No difference was observed between G12 (m-sham-ad) and G9 (m-orx-ad) nor between G12 (m-sham-ad) and G11 (m-sham-iso). These groups (G9, G11, and G12) had the highest values of Ca/P.

For both females and males, all groups with the alcoholic diet (G1, G4, G7, and G10) had a lower Ca/P compared to groups with the* ad libitum* diet (G3, G6, G9, and G12). The lowest values of Ca/P were found in groups subjected to the alcoholic diet in conjunction with hormonal deficiency: G1 (f-ovx-alc) for females and G7 (m-orx-alc) for males. These groups were used to test the hypothesis of this study.

## 4. Discussion

Our results suggest that diet had an important role, since the groups with* ad libitum* diet gained more weight in both females and males. However, factors related to gender also seem to have influenced the results, since all females gained weight during the experiment but some males showed weight reduction. This gender difference was previously observed by Jack Wallen et al. [25], who noticed that the deficiency of sex hormones reduced weight in males but increased it in females. Our study also showed that weight gain (in absolute values) was even more evident in females with estrogen deficiency (Ovx) and* ad libitum* diet (G3). This is similar to what occurs in women with menopause when high prevalence of overweight/obesity is reported [26].

For males, feed efficiency values were proportional to caloric intake for all groups. On the other hand, females on the alcoholic diet presented high caloric intake but low feed efficiency. One possible explanation for these results could be the great difficulty females have in metabolizing alcohol. Female bodies have lower total water volume compared to males. Because of this, females present higher blood alcohol levels than males given the same alcohol consumption [27]. In addition, females present lower gastric metabolism with lower alcohol dehydrogenase (ADH) activity [28], as well as a reduction of gluconeogenesis [29].

The Ca/P ratio seems to be more reliable for analysis of bone mineralization disorders than separate analyses of Ca and P [30]. In experiments that induce osteoporosis, a decrease in Ca/P is expected compared to normal bone [4]. Despite the well-known negative effect of the lack of sex hormones on bone quality [7, 9], there was no alteration in the Ca/P ratio when the sex hormone deficiency was assessed alone (without association with dietary restrictions), in the present study. This was verified by the results observed in* ad libitum* groups in which sex hormone deficiency did not alter Ca/P ratio in both females and males; group G3 (f-ovx-ad) was not different from G6 (f-sham-ad), and G9 (m-orx-ad) was not different from G12 (m-sham-ad). However, the conjunction of sex hormone deficiency and alcoholic diet (G1 and G7) led to the lowest Ca/P ratios for both females and males, which confirmed the initial hypothesis of our experiment. A previous study [19] observed a decrease of the Ca/P ratio in the alveolar bone crest region of Ovx females receiving a 20% alcohol solution for 8 weeks (the same experimental protocol of this study). In another study, Callaci et al. [18] observed vertebral bone loss in Ovx females receiving alcohol. This suggests that the deficiency of sex hormones associated with alcohol can affect different types of bones and is not restricted to the femur.

When comparing to groups with* ad libitum* diets, we observed a decreased Ca/P ratio in all groups that ingested alcohol and in the majority of isocaloric groups, with the exception of the male group G11 (m-sham-iso), in which the highest Ca/P ratio was found. These results suggest that the decrease in the Ca/P ratio was evident for both females and males with an alcoholic diet. On the other hand, for groups with the isocaloric diet, the decrease in Ca/P ratio was more evident in females.

The use of isocaloric groups aimed to control for the nutritional problems related to a lower intake of food. Studies show that rats treated with alcohol ingest a significantly lower amount of solid food compared to groups with* ad libitum* diets [19, 21, 22]. This happens because alcohol is a substance with a low nutritional value but high energy content, which may lead to a feeling of satiety [15]. The use of isocaloric groups allows a control for the possible influence of decreased food intake on the results. It should be considered that an adequate intake of nutrients is important for the prevention and management of osteoporosis [16].

However, alcohol causes other important metabolic changes that were not controlled in this experiment. Alcohol consumption can lead to changes in the mucosa of the gastrointestinal tract, increased transport of toxins, and maldigestion and malabsorption of nutrients [31]. Alcohol also alters the metabolism of nutrients since it is a toxic substance which cannot be stored, and so alcohol must be oxidized preferentially to other nutrients [32]. Furthermore, alcohol causes various hormonal disorders involving secretions of several glands such as the hypothalamus, anterior pituitary, adrenal cortex, testicles, ovaries, thyroid gland, and pancreas, which could cause physiological and behavioral disorders [33]. The literature also reports that excessive alcohol consumption can lead to a decrease in vitamin D levels [34] which could impair calcium absorption [35]. It has been suggested that low levels of vitamin D in combination with low levels of sex steroid hormones considerably increase the risk of bone fractures [36].

In addition to the indirect effects of alcohol on bone metabolism causing important nutritional and hormonal changes, alcohol can also directly influence the activity of bone cells. The decrease in bone mass induced by alcohol consumption can be attributed to an imbalance between bone formation and bone resorption, with a predominant decrease in bone formation [13]. Rosa et al. [14] observed that alcohol intake affected the osteoblast function (important cells for bone formation and mineralization) in cell culture, inhibiting the bone matrix mineralization process. Similarly, a change in the pattern of bone mineralization (characterized by a decrease in the Ca/P ratio) was observed in animals of alcohol groups (when compared to* ad libitum* groups), in the present experiment.

We observed that the alcoholic diet groups showed a state of lethargy and excessive rest, which may also have influenced the results since exercise can have a preventive effect on bone loss [37]. Therefore, it is not possible to know whether the results obtained in the alcohol groups are due to metabolic effects of alcohol, the state of lethargy and excessive resting (decreasing the physical activity), or both.

Another factor that must be considered is that the alcohol groups ingested the least amount of water, since dehydration could theoretically cause changes in the structure of hydroxyapatite [38, 39].

Some differences between males and females should be emphasized. The decrease of the Ca/P ratio found in Orx males subjected to alcohol consumption (G7) was influenced mainly by a significant increase in phosphorus. On the other hand, in Ovx females subjected to alcohol consumption (G1) the decrease of the Ca/P ratio was influenced mainly by a significant decrease in Ca. The literature reports that both Ca and P are important nutrients for maintaining bone health [16]. However, it has also been suggested that an excess of phosphorus can impair bone health and be a risk factor for fractures [40].

This study has some limitations. The bone analysis is related only to its cortical area, since the X-ray coming from the rhodium tube does not penetrate deep into the sample. Therefore, there was no data related to trabecular bone. The loss of cortical and trabecular bone is important variable in assessing the risk of osteoporotic fractures [41]. An evaluation of trabecular bone could be considered in future studies.

In this study, only Ca and P were directly measured by the equipment. The organic elements were not directly measured because the equipment used in this study was not able to perform measurements for chemical elements below Na in atomic number. This fact can be considered a limitation of this study, since the bone inorganic phase consists of poorly crystalline carbonated apatite [1, 42]. Minerals are the main components of bone tissue, but not the only ones. Bone tissue is composed of minerals (50–70%), organic matrix (20–40%), water (5–10%), and lipids (<3%) [1]. A disease like osteoporosis is complex and can affect mineral and nonmineral components [43]. Other techniques such as Infrared and Raman spectroscopy could provide qualitative and quantitative information about the mineral and organic components of bone tissue and could be used in future studies [44].

It was previously suggested that, after comparison with other studies, the intake of alcohol by rats at a concentration of 20% for 8 weeks led to alcohol levels similar to that of animals having chronic and excessive alcohol consumption [19]. However, this comparison should be viewed with caution, since it would necessitate assessments of blood alcohol levels to have reliability. The lack of assessment of blood alcohol levels can be considered another limitation of the present study.

## 5. Conclusions

The present study demonstrated that 8 weeks of consuming 20% alcoholic solution reduced the Ca/P ratio in the femurs of both female and male rats. The isocaloric diet reduced the Ca/P ratio in females. In groups with* ad libitum* diet, the deficiency of sex hormones did change the Ca/P ratio in females and males. However, the deficiency of sex hormones in conjunction with an alcoholic diet promoted the lowest values for the Ca/P ratio in both females and males.

In short, one can conclude that there was a reduction in the Ca/P ratio in the femur of both male and female rats who consumed alcohol and that this reduction was exacerbated when combined with a deficiency of sex hormones.

## Figures and Tables

**Table 1:** Description of the experimental groups.

Group number	Group name (short form)	Gender	Surgery	Diet
G1	f-ovx-alc	Female	Ovariectomy	Alcoholic
G2	f-ovx-iso	Female	Ovariectomy	Isocaloric
G3	f-ovx-ad	Female	Ovariectomy	*Ad libitum*
G4	f-sham-alc	Female	Sham	Alcoholic
G5	f-sham-iso	Female	Sham	Isocaloric
G6	f-sham-ad	Female	Sham	*Ad libitum*
G7	m-orx-alc	Male	Orchiectomy	Alcoholic
G8	m-orx-iso	Male	Orchiectomy	Isocaloric
G9	m-orx-ad	Male	Orchiectomy	*Ad libitum*
G10	m-sham-alc	Male	Sham	Alcoholic
G11	m-sham-iso	Male	Sham	Isocaloric
G12	m-sham-ad	Male	Sham	*Ad libitum*

**Table 2:** Total caloric intake, alcohol intake, water, and feed efficiency.

Group	Total caloric intake (kcal)	Alcohol intake (kcal)	Water (mL)	Feed efficiency (%)
G1 (f-ovx-alc)	64.31	18.40	12.99	21.61
G2 (f-ovx-iso)	58.31	—	27.13	29.65
G3 (f-ovx-ad)	66.19	—	25.0	46.61
G4 (f-sham-alc)	65.70	19.41	13.71	24.25
G5 (f-sham-iso)	57.76	—	28.24	19.22
G6 (f-sham-ad)	60.37	—	32.00	33.03
G7 (m-orx-alc)	70.11	20.71	16.57	−3.85
G8 (m-orx-iso)	69.32	—	36.19	−4.73
G9 (m-orx-ad)	90.34	—	39.70	10.23
G10 (m-sham-alc)	80.93	19.63	15.70	8.11
G11 (m-sham-iso)	72.17	—	33.90	−3.33
G12 (m-sham-ad)	105.83	—	43.04	17.08
